# Multistate survival modelling of multimorbidity and transitions across health needs states and death in an ageing population

**DOI:** 10.1136/jech-2023-220570

**Published:** 2024-01-11

**Authors:** Qian Gao, Graciela Muniz Terrera, Rosie Mayston, Matthew Prina

**Affiliations:** 1 Department of Behavioural Science and Health, Institute of Epidemiology & Health Care, University College London, London, UK; 2 Health Service and Population Research, Institute of Psychiatry, Psychology & Neuroscience, King's College London, London, UK; 3 Centre for Clinical Brain Sciences, The University of Edinburgh, Edinburgh, UK; 4 Global Health & Social Medicine & King’s Global Health Institute, Social Science & Public Policy, King's College London, London, UK; 5 Population Health Sciences Institute, Faculty of Medical Sciences, Newcastle University, Newcastle upon Tyne, UK

**Keywords:** AGING, MORTALITY, HEALTH SERVICES, HEALTH STATUS, EPIDEMIOLOGY

## Abstract

**Background:**

Unmet health needs have the potential to capture health inequality. Nevertheless, the course of healthcare needs fulfilment, and the role of multimorbidity in this process remains unclear. This study assessed the bidirectional transitions between met and unmet health needs and the transition to death and examined the effect of multimorbidity on transitions.

**Methods:**

This study was based on the China Health and Retirement Longitudinal Study, a nationally representative survey in 2011–2015 among 18 075 participants aged 45 and above (average age 61.1; SD 9.9). We applied a multistate survival model to estimate the probabilities and the instantaneous risk of state transitions, and Gompertz hazard models were fitted to estimate the total, marginal and state-specific life expectancies (LEs).

**Results:**

Living with physical multimorbidity (HR=1.85, 95% CI 1.58 to 2.15) or physical–mental multimorbidity (HR=1.45, 95% CI 1.15 to 1.82) was associated with an increased risk of transitioning into unmet healthcare needs compared with no multimorbidity. Conversely, multimorbidity groups had a decreased risk of transitioning out of unmet needs. Multimorbidity was also associated with shortened total life expectancy (TLEs), and the proportion of marginal LE for having unmet needs was more than two times higher than no multimorbidity.

**Conclusion:**

Multimorbidity aggravates the risk of transitioning into having unmet healthcare needs in the middle and later life, leading to a notable reduction in TLEs, with longer times spent with unmet needs. Policy inputs on developing integrated person-centred services and specifically scaling up to target the complex health needs of ageing populations need to be in place.

**WHAT IS ALREADY KNOWN ON THIS TOPIC**

- Unmet health needs measure the gap between services needed in theory and the extent received, which has been recognised as an important indicator of health inequality. As populations age, the increasingly prevalent multiple chronic conditions may further complicate the diverse and long-term health needs of older adults. Yet, many unknowns remain about the course of health needs fulfilment and the relationship with multimorbidity.

**WHAT THIS STUDY ADDS**

- In this population-based cohort study involving 18 075 middle-aged and older adults in China, we found that multimorbidity was longitudinally associated with an increased risk of transitioning into unmet healthcare needs; in turn, the transition out of unmet needs was attenuated by multimorbidity. Marginal life expectancies for having unmet healthcare needs were predicted to account for larger proportions of the overall life expectancy for those living with physical or physical–mental multimorbidity than no multimorbidity.

**HOW THIS STUDY MIGHT AFFECT RESEARCH, PRACTICE OR POLICY**

- The findings highlight the crucial role of multimorbidity in aggravating the onset and hindering the transition out of unmet healthcare needs, supporting the necessity of developing and scaling up personal-centred comprehensive health service packages to address complex health needs derived from physical–mental multimorbidity.

## Introduction

Evidence suggests that the healthcare needs of older adults are often poorly served by health services, particularly among those who have more health difficulties.^1 2^ Inequitable access to health services is still present in most health systems, especially in low-income and middle-income countries (LMICs), including China, leading to unmet needs.^2^ Unmet healthcare needs measure the gap between the healthcare services necessary to manage health problems and the extent received in reality,^3^ highlighting potential misalignments between the health system offering and population needs. It is a potential approach for monitoring the performance of health systems in terms of whether what is available meets the needs and preferences of the population, providing a more holistic estimate of equity of health service provision compared with individual measures of healthcare utilisation.^4 5^ Theoretical evidence has been yielded in terms of conceptualising different categories of unmet healthcare needs, for example, unperceived, subjectively chosen or not chosen, clinician-validated unmet needs, and unmet expectations of healthcare.^4^ Previous epidemiological research has commonly adopted practical measures of unmet healthcare needs by taking account of the ‘need’ factor in actual healthcare utilisation, which has identified a wide range of correlates of unmet healthcare needs, including older age, lower socioeconomic status, physical and psychological health conditions,^6–8^ suggesting unmet needs are an indicator of disadvantage.

Multimorbidity is defined as two or more copresent chronic physical and mental health conditions, emerging as one of the major challenges facing ageing societies.^9^ There is evidence that multimorbidity is associated with socioeconomic deprivation,^9^ functional limitations^10^ and greater healthcare use.^11 12^ Given an increasing life expectancy across the globe, including in China, the rise in multimorbidity will continue to burden fragmented healthcare systems, especially in resource-limited settings.^13–15^ In our previous research, we found that unmet healthcare needs are more concentrated among people living with multimorbidity, with barriers in low affordability and acceptability and perceived inadequacy of health services.^8^ Chinese older adults with physical multimorbidity have also been found to be at greater risk of having catastrophic health expenditures, regardless of their socioeconomic status and enrolment in health insurance programmes.^16^

To date, most studies investigating unmet healthcare needs and the relationship with multimorbidity have a cross-sectional design. Therefore, important evidence about the role of multimorbidity in triggering the onset/out of unmet needs is missing; likewise, we do not understand its impacts on the links between unmet needs and death. It has been suggested that experiencing unmet healthcare needs is closely linked with barriers to accessing health services: due to the issues at an individual level (eg, socioeconomic disadvantages, health beliefs about healthcare and diseases, etc) and to gaps between health and social protection systems (eg, barriers in accessibility, affordability, perceived inadequacy of healthcare, etc),^4 8^ resulting in unmet health needs that may fluctuate over time. It seems plausible that having persistent unmet healthcare needs may worsen downstream health outcomes. Conversely, as the number of older individuals who have greater needs for care is growing, there is an inevitable growth in healthcare needs that are not necessarily addressed within current infrastructures (family and societal support) and health systems. Understanding the course of unmet needs, in terms of when these emerge, whether multimorbidity is associated with emergence and remission, and the effect on mortality potentially provides a comprehensive capture of the barriers to universal health coverage (UHC) and has important implications for tackling health inequalities. The study aims: (1) to calculate the probability of making transitions: (a) from met to unmet healthcare needs; (b) from unmet needs to death; and (c) returning from unmet needs to met healthcare needs; (2) to investigate the effect of multimorbidity on these transitions; (3) to estimate the total life expectancies (TLEs) for older adults, LEs among those having met/unmet healthcare needs, and to estimate the effects of multimorbidity on each of these.

## Methods

### Data resources and sample

The China Health and Retirement Longitudinal Study (CHARLS) survey provides comprehensive data on the health, social and economic conditions of community residents aged 45 years old and above, using a multistage probability sampling approach. The study included three waves of CHARLS. Individuals have been followed up every 2 years, with the non-response baseline samples retracing in follow-ups. The baseline wave (in 2011) had a response rate of 80.5%, consisting of 17 708 respondents from 10 000 households in 150 counties/districts across 28 provinces. Follow-up rates were 82.6% and 82.1% for wave 2 in 2013 (n=18 264) and wave 3 in 2015 (n=20 284), respectively. Details of the cohort have been described elsewhere.^17^ In our sample, we included participants who had information on health needs states at baseline and those with vital status at the end of wave 2/3 (n=18 075). Informed consent was obtained from all participants.

### Measures

#### Outcome

Healthcare needs fulfilment (met/unmet) was assessed by combining two measurements on whether participants had unmet needs for inpatient or outpatient care: (1) participants were classified as having unmet needs for outpatient care if they were ill (measured by self-reported getting ill in the last month) but did not use any outpatient care (eg, public hospital, private hospital, public health centre, clinic, or health worker’s or doctor’s practice, or home visit by a health worker or doctor for outpatient care) in the past month; (2) participants were categorised as having unmet needs for inpatient care if, in the past year, a doctor suggested that they needed inpatient care, but did not get hospitalised. Overall, participants were categorised as having unmet healthcare needs if they had unmet needs for either inpatient or outpatient care. Those who met inpatient and outpatient care needs were classified as met healthcare needs. The same approach was used to determine healthcare needs fulfilment (met/unmet) at all waves. This assessment has been applied in previous research^8^ to conceptualise unmet health needs.^4 18^

#### Covariates

Information on age, sex and education was included in the analyses. The physician-diagnosed chronic disorders were self-reported through a list of 14 physical and mental illnesses (online supplemental table S1). We also considered depression in this study, which was measured by the 10-item Centre for Epidemiological Studies Depression Scale, capturing the frequency of participants’ experiences of various symptoms included on the scale in the past week. Responses to individual items were scored from 0 to 3, with a total score ranging from 0 to 30. We used a cut-off score≥10 to categorise depression.^19^ Finally, the 15 chronic health conditions mentioned above were classified into a categorical variable multimorbidity: physical-mental multimorbidity (PM-multimorbidity)/physical multimorbidity (P-multimorbidity)/none. This was defined as having two or more conditions in each category of multimorbidity.^9^

10.1136/jech-2023-220570.supp1 Supplementary data

### Statistical analysis

Multistate modelling (MSM) based on the Markov assumption was used to simultaneously estimate the transition intensities across states by adopting a three-state model (a pictorial representation of the fitted three-state model, in online supplemental figure S1). We initially conducted multistate survival analyses to estimate the transition distribution, transition probabilities and the instantaneous risk of moving across three states. We also wanted to simultaneously test the role of covariates (time-varying age and multimorbidity, sex and education) in (1) transition into unmet healthcare needs (new case); (2) transition out of unmet needs (remission); (3) transition from met healthcare needs to death; (4) transition from unmet healthcare needs to death, presenting the HRs and 95% CIs for each. The model fit was estimated by the Akaike information criterion. We took account of interval-censoring and right-censoring (alive but with an unknown last state) in all MSM estimates. For those respondents who were known to have died but had missingness in the date of death in follow-up surveys, only the vital status was used as there was no information on the date of death. We then imputed the missing date of death using generated random dates between each corresponding interviewing wave and the previous one. The MSM package for R was applied for all computations.^20 21^

For LEs estimation, we fitted new parametric models (Gompertz hazard models) in MSM by including time-independent covariates (sex, education and baseline multimorbidity) instead of the initial MSM. Total residual life expectancy (TLE), marginal and state-specific LEs were calculated based on the maximum likelihood point estimate of model parameters in the fitted Gompertz hazard model using piecewise-constant approximation.^22^ TLE and marginal LEs for unmet and met healthcare needs were calculated in the overall sample and by sex, education and baseline multimorbidity at different estimated ages. We then calculated the state-specific LEs for having unmet (state 1)/met healthcare needs (state 2) across covariates by different starting states at estimated ages. All estimates of LEs were conducted using the ELECT (Estimating Life Expectancies in Continuous Time, Version 0.2) package for R.^22 23^ A simulation-based approach was adopted to provide 95% CI for the estimated LEs.^24^ The max-age was set at 115 years in all computations of LEs, aligning with the age range of the cohort (45–102.4 years). All the analyses were conducted using Stata, V.17.0 (StataCorp LLC) and R Studio, V.1.4.1103 (R Project for Statistical Computing).

## Results

### Study samples and the transition distribution

A total of 18 075 participants (mean age 61.1 years, SD=9.9) were included in the transition analyses, among whom 50.9% were female, and 67.0% had lower levels of education. 8.3% of the sample had PM-multimorbidity, and 45.6% of the participants had P-multimorbidity. The characteristics of samples across waves are presented in online supplemental table S2. Across the follow-up, 995 cases and 11 151 cases persistently reported unmet/met healthcare needs in two consecutive waves. There were 2067 people transitioning into unmet needs and 1719 transitioning out of unmet needs. There were 122 cases who transitioned from unmet needs to death, and 440 observations of the transition from met needs to death (online supplemental table S3).

### Transition probabilities and effects of covariates on transitions

The 2-year transition probability for transitioning into unmet healthcare needs was predicted to be highest among those with P-multimorbidity 0.201 (95% CI 0.186 to 0.217), followed by PM-multimorbidity 0.155 (95% CI 0.136 to 0.176), whereas it was lowest among participants without multimorbidity 0.089 (95% CI 0.082 to 0.096). In turn, transition out of unmet needs was more likely to be found in the group without multimorbidity 0.780 (95% CI 0.742 to 0.808). The probability of having persistent unmet needs in two consecutive waves was predicted to be higher among those living with P-multimorbidity 0.419 (95% CI 0.389 to 0.452) and PM-multimorbidity 0.361 (95% CI 0.306 to 0.419). The 2-year transition probabilities for transitioning to death were higher for the unmet needs group compared with the group whose needs were met among those with multimorbidity (table 1).

**Table 1:** The 2-year transition probability for transitioning across states by multimorbidity

Transitions	Transition probabilities		
	No multimorbidity	P-multimorbidity	PM-multimorbidity
Unmet health needs → Met health needs	0.780 (0.742 to 0.808)	0.555 (0.522 to 0.586)	0.608 (0.545 to 0.666)
Me health needs → Unmet health needs	0.089 (0.082 to 0.096)	0.201 (0.186 to 0.217)	0.155 (0.136 to 0.176)
Unmet health needs → Death	0.011 (0.008 to 0.025)	0.026 (0.019 to 0.039)	0.031 (0.018 to 0.064)
Met health needs→ Death	0.013 (0.011 to 0.016)	0.022 (0.019 to 0.027)	0.019 (0.015 to 0.025)
Met needs in two consecutive waves	0.899 (0.891 to 0.906)	0.777 (0.762 to 0.792)	0.826 (0.803 to 0.846)
Unmet needs in two consecutive waves	0.209 (0.180 to 0.243)	0.419 (0.389 to 0.452)	0.361 (0.306 to 0.419)

In the estimates, covariates (including age, sex and education) were set to the mean of dummy variables. Model adjusted for age, sex, education and time-varying multimorbidity.

Given the results of multistate survival modelling, the effects of covariates on transitions across the states of healthcare needs and death are shown in table 2. Increasing age and being male were associated with a greater risk of transitioning from both met/unmet needs to death (5-year age effect on transitions, in online supplemental figure S2). Females were at increased risk of transitioning into unmet needs (HR=1.28, 95% CI 1.11 to 1.47). Higher education level had a protective effect on transitioning from met healthcare needs to death, and it also increased the probability of remission. Compared with those without multimorbidity, the group with PM-multimorbidity (HR=1.45, 95% CI 1.15 to 1.82) and those living with P-multimorbidity (HR=1.85, 95% CI 1.58 to 2.15) had higher risks of transitioning into unmet healthcare needs. Conversely, the transition out of unmet needs was attenuated by multimorbidity. P-multimorbidity was associated with an increased risk of transitioning from met healthcare needs to death (HR=1.68, 95% CI 1.34 to 2.11); however, statistical evidence on the effects of multimorbidity on transitioning from unmet needs to death was weak.

**Table 2:** HRs (95% CI) for the effect of covariates on transitions across states of met and unmet healthcare needs and death

Covariates	Transition I *Transition out of unmet needs (remission*)		Transition II *Transition into unmet needs (progression*)	
	Unadjusted HRs	Adjusted HRs*	Unadjusted HRs	Adjusted HRs*
Age	1.00 (1.00 to 1.01)	1.01 (1.00 to 1.02)	1.00 (1.00 to 1.01)	1.00 (0.99 to 1.01)
Female (ref. male)	1.02 (0.91 to 1.15)	1.11 (0.97 to 1.26)	1.32 (1.17 to 1.50)	1.28 (1.11 to 1.47)
Middle education (ref. low)	1.09 (0.94 to1.27)	1.13 (0.95 to 1.35)	0.85 (0.72 to 1.00)	0.95 (0.79 to 1.15)
High education (ref. low)	1.34 (1.08 to 1.67)	1.31 (1.02 to 1.67)	1.10 (0.87 to 1.38)	1.20 (0.93 to1.55)
P-multimorbidity (ref. no)	0.57 (0.49 to 0.66)	0.58 (0.50 to 0.67)	1.85 (1.59 to 2.15)	1.85 (1.58 to 2.15)
PM-multimorbidity (ref. no)	0.63 (0.51 to 0.79)	0.64 (0.52 to 0.80)	1.46 (1.16 to 1.84)	1.45 (1.15 to 1.82)
	**Transition III** ***Unmet healthcare needs*** **→** ***Death***		**Transition IV** ***Met healthcare needs*** **→** ***Death***	
	**Unadjusted HRs**	Adjusted HRs*	**Unadjusted HRs**	Adjusted HRs*
Age	1.09 (1.06 to 1.13)	1.05 (1.01 to 1.09)	1.11 (1.09 to 1.12)	1.11 (1.10 to 1.12)
Female (ref. male)	0.63 (0.31 to 1.28)	0.42 (0.20 to 0.88)	0.78 (0.61 to 1.00)	0.79 (0.64 to 0.97)
Middle education (ref. low)	1.77 (0.77 to 4.10)	2.12 (0.99 to 4.53)	0.99 (0.68 to 1.46)	0.80 (0.53 to 1.20)
High education (ref. low)	0.85 (0.17 to 4.31)	1.20 (0.44 to 3.29)	0.57 (0.29 to 1.11)	0.41 (0.22 to 0.77)
P-multimorbidity (ref. no)	4.85 (0.62 to 37.84)	2.81 (0.75 to10.46)	1.56 (1.24 to 1.95)	1.68 (1.34 to 2.11)
PM-multimorbidity (ref. no)	5.92 (0.68 to 51.80)	3.99 (0.94 to 16.88)	1.32 (0.90 to 1.95)	1.30 (0.88 to 1.91)

High-education: high school and above; middle-education: middle school; low-education: elementary school and below.

*The estimates were based on multistate survival model. Final model adjusted for age, sex, education and time-varying multimorbidity (model fit: −2loglik=22 514.77; AIC=22 570.77).

PM-multimorbidity, physical-mental multimorbidity; P-multimorbidity, physical multimorbidity.

### Total, marginal and state-specific life expectancy

**Figure 1:**
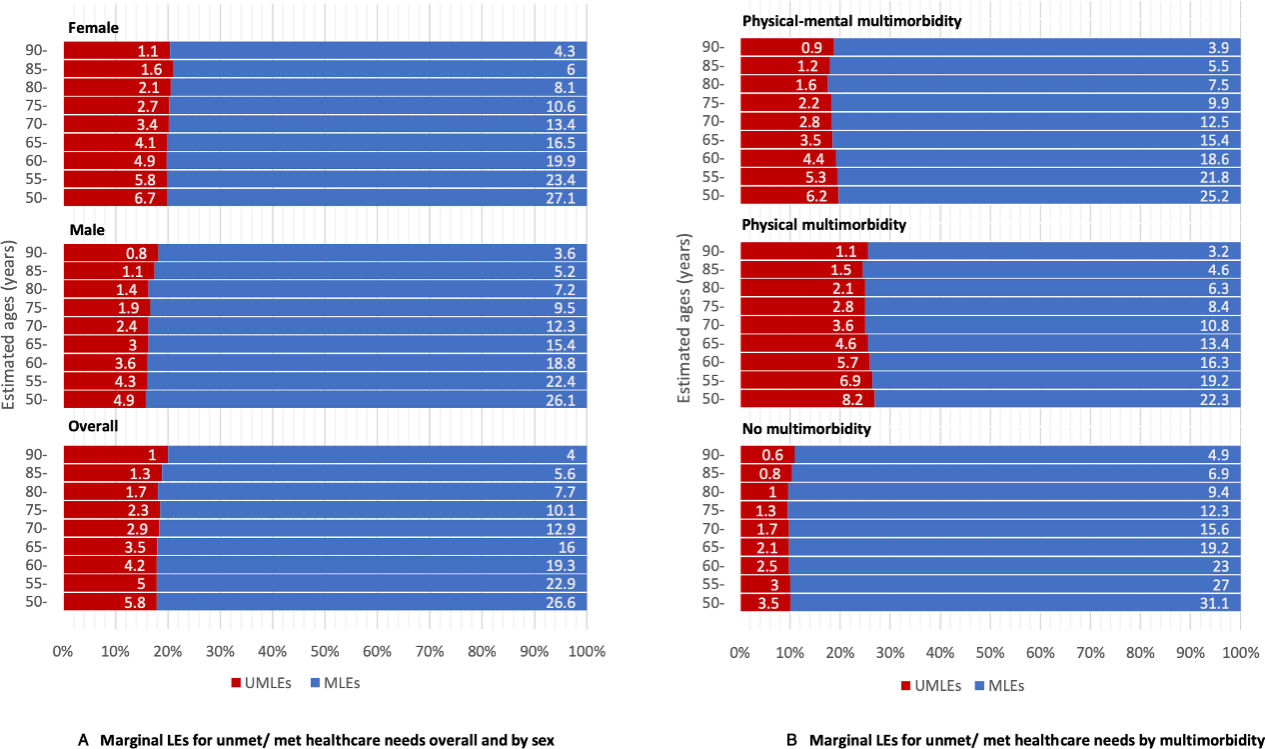
Marginal life expectancies in years for having unmet/met healthcare needs at different estimated ages (50–90 years) by sex (A) and multimorbidity (B). MLEs, marginal life expectancies for met healthcare needs; UMLEs, marginal life expectancies for unmet healthcare needs.

**Figure 2:**
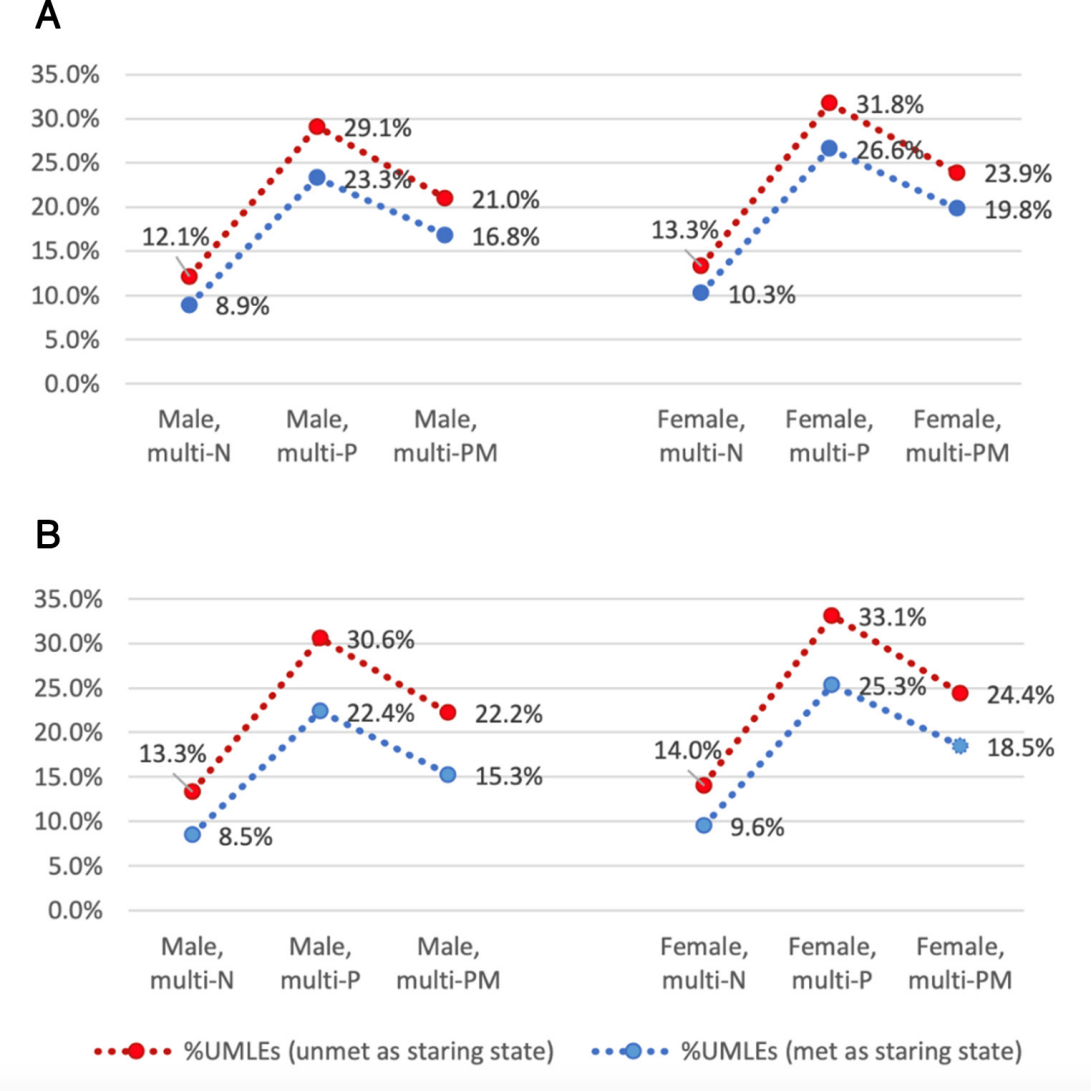
Proportions of years spent with having unmet healthcare needs over total life expectancies (TLEs) with different starting states by sex and multimorbidity at age 60 (A) and at age 70 (B). multi-N, no multimorbidity; multi-P, physical multimorbidity; multi-PM, physical–mental multimorbidity. %UMLEs refers to the proportion of marginal life expectancy for having unmet healthcare needs over TLE.

The estimates of LEs for participants with different sex, educational attainment and baseline multimorbidity are displayed in table 3 (results for MSM, in online supplemental table S4). Overall, LE at age 60 was 23.6 (95% CI 22.8 to 24.3) years, with 4.2 years spent having unmet needs, accounting for 17.8% of TLE. TLE was predicted to be higher for women (24.8 years (95% CI 23.6 to 25.8)) than men, but with women spending more time with unmet needs (4.9 years, 19.8% of TLE) than men (3.6 years, 16.1% of TLE) at age 60. The multimorbidity group had the shortest TLEs (22.0 years (95% CI 20.9 to 22.8) for physical-multimorbidity and 22.9 years (20.8–24.7) for physical–mental multimorbidity). The proportion of marginal LEs for having unmet needs was greater in those with physical or physical–mental multimorbidity (5.7 years (5.3–6.1), 25.9% of TLE and 4.4 years (3.7–5.0), 19.2% of TLE, respectively). The gaps in TLEs and marginal LEs between those with multimorbidity and no multimorbidity were consistent across all estimated age groups (50–90 years). The proportion of marginal LEs for having unmet needs raised slightly with increasing age in the no multimorbidity group, but the trend was inverse in those with multimorbidity (figure 1). Years spent with having unmet needs were consistently higher in those who had unmet needs as a starting state, accounting for larger proportions in the corresponding estimated TLEs. The differences led by starting states were consistent across sex, education and multimorbidity status, with the gap being larger at an older estimated age (figure 2).

**Table 3:** Total, marginal and state-specific life expectancies for participants by sex, education and baseline multimorbidity (in years, 95% CI)

Age 60	TLEs*	Marginal LEs		%UMLEs†	LEs in having unmet health needs by different starting state		LEs in having health needs being met by different starting state	
		Unmet health needs	Met health needs		Unmet health needs‡	Met health needs§	Unmet health needs‡	Met health needs§
Overall	23.6 (22.8 to 24.3)	4.2 (4.0 to 4.5)	19.3 (18.7 to 19.9)	17.8	5.2 (4.9 to 5.5)	4.1 (3.8 to 4.3)	18.3 (17.6 to 18.9)	19.5 (18.8 to 20.1)
Sex								
Male	22.3 (21.4 to 23.3)	3.6 (3.3 to 3.9)	18.8 (18.0 to 19.6)	16.1	4.6 (4.2 to 4.9)	3.4 (3.1 to 3.7)	17.7 (16.9 to 18.5)	19.0 (18.2 to 19.8)
Female	24.8 (23.6 to 25.8)	4.9 (4.5 to 5.3)	19.9 (18.8 to 20.7)	19.8	5.9 (5.5 to 6.3)	4.8 (4.4 to 5.1)	18.9 (17.9 to 19.7)	20.0 (19.0 to 20.8)
Education								
Low	23.5 (22.6 to 24.1)	4.3 (4.0 to 4.6)	19.1 (18.4 to 19.8)	18.3	5.3 (5.0 to 5.6)	4.1 (3.8 to 4.4)	18.1 (17.4 to 18.7)	19.3 (18.6 to 20.0)
Middle	22.6 (20.3 to 24.3)	3.3 (2.8 to 3.7)	19.3 (17.3 to 20.8)	14.6	4.2 (3.8 to 4.7)	3.1 (2.7 to 3.5)	18.1 (16.1 to 19.6)	19.5 (17.5 to 21.0)
High	27.7 (23.2 to 30.6)	4.3 (3.5 to 5.0)	23.4 (19.6 to 26.1)	15.5	5.1 (4.2 to 5.7)	4.2 (3.3 to 4.8)	22.6 (18.7 to 25.2)	23.6 (19.7 to 26.2)
Multimorbidity								
multi-N	25.5 (24.2 to 26.8)	2.5 (2.3 to 2.8)	23.0 (21.8 to 24.1)	9.8	3.2 (3.0 to 3.5)	2.4 (2.2 to 2.7)	22.3 (21.1 to 23.4)	23.1 (21.9 to 24.2)
multi-P	22.0 (20.9 to 22.8)	5.7 (5.3 to 6.1)	16.3 (15.5 to 16.9)	25.9	6.7 (6.2 to 7.1)	5.5 (5.1 to 5.9)	15.2 (14.4 to 15.9)	16.5 (15.7 to 17.1)
multi-PM	22.9 (20.8 to 24.7)	4.4 (3.7 to 5.0)	18.6 (16.8 to 20.2)	19.2	5.2 (4.6 to 5.8)	4.2 (3.6 to 4.8)	17.5 (15.6 to 18.9)	18.7 (16.9 to 20.3)
Age 60, by sex and multimorbidity								
Male, multi-N	24.7 (23.3 to 25.9)	2.3 (2.1 to 2.5)	22.4 (21.1 to 23.5)	9.3	3.0 (2.8 to 3.3)	2.2 (2.0 to 2.4)	21.7 (20.2 to 22.8)	22.5 (21.2 to 23.6)
Male, multi-P	20.6 (19.4 to 21.5)	5.0 (4.6 to 5.4)	15.6 (14.7 to 16.3)	24.3	6.0 (5.5 to 6.4)	4.8 (4.4 to 5.2)	14.4 (13.5 to 15.1)	15.8 (15.0 to 16.5)
Male, multi-PM	21.4 (19.1 to 23.4)	3.7 (3.1 to 4.2)	17.7 (15.8 to 19.5)	17.3	4.5 (3.9 to 5.1)	3.6 (3.0 to 4.1)	16.3 (14.2 to 18.3)	18.0 (16.0 to 19.8)
Female, multi-N	26.3 (24.7 to 27.8)	2.8 (2.5 to 3.1)	23.5 (22.2 to 24.8)	10.6	3.5 (3.1 to 3.8)	2.7 (2.4 to 3.0)	22.9 (21.5 to 24.1)	23.6 (22.3 to 24.9)
Female, multi-P	23.3 (21.9 to 24.3)	6.4 (5.9 to 7.0)	16.9 (15.9 to 17.6)	27.5	7.4 (6.8 to 7.9)	6.2 (5.7 to 6.8)	15.9 (14.9 to 16.7)	17.0 (16.0 to 17.8)
Female, multi-PM	24.7 (22.4 to 26.9)	5.0 (4.3 to 5.7)	19.7 (17.8 to 21.5)	20.2	5.9 (5.1 to 6.6)	4.9 (4.1 to 5.5)	18.7 (16.9 to 20.6)	19.9 (18.0 to 21.7)
Age 70, by sex and multimorbidity								
Male, multi-N	16.5 (15.2 to 17.7)	1.5 (1.3 to 1.7)	15.0 (13.8 to 16.1)	9.1	2.2 (1.9 to 2.4)	1.4 (1.2 to 1.6)	14.4 (13.2 to 15.5)	15.1 (13.9 to 16.2)
Male, multi-P	13.4 (12.3 to 14.2)	3.1 (2.8 to 3.5)	10.2 (9.4 to 10.9)	23.1	4.1 (3.7 to 4.4)	3.0 (2.6 to 3.3)	9.2 (8.3 to 9.9)	10.4 (9.6 to 11.1)
Male, multi-PM	14.4 (12.5 to 16.3)	2.4 (2.0 to 2.8)	12.0 (10.3 to 13.5)	16.7	3.2 (2.6 to 3.6)	2.2 (1.8 to 2.6)	10.7 (9.2 to 12.3)	12.2 (10.6 to 13.8)
Female, multi-N	17.8 (16.5 to 19.1)	1.9 (1.6 to 2.1)	15.9 (14.7 to 17.1)	10.7	2.5 (2.2 to 2.8)	1.7 (1.5 to 2.0)	15.5 (14.3 to 16.7)	16.0 (14.8 to 17.2)
Female, multi-P	15.4 (14.1 to 16.4)	4.1 (3.7 to 4.5)	11.2 (10.3 to 12.0)	26.6	5.1 (4.6 to 5.5)	3.9 (3.5 to 4.4)	10.5 (9.6 to 11.3)	11.4 (10.5 to 12.2)
Female, multi-PM	16.8 (14.7 to 18.9)	3.3 (2.7 to 3.9)	13.5 (11.7 to 15.2)	19.6	4.1 (3.5 to 4.7)	3.1 (2.6 to 3.7)	12.7 (11.0 to 14.4)	13.7 (11.9 to 15.4)

Estimates were based on multistate survival models and separately adjusted for age, sex, education, and baseline multimorbidity at ages 60 and 70.

*All estimates refer to life expectancies at the corresponding estimated age.

†%UMLEs refer to the proportion of marginal life expectancy for having unmet healthcare needs over total life expectancy.

‡Refers to life expectancies in having unmet/met needs for those in state 1 (unmet health needs) at the estimated age.

§Refers to life expectancies in having unmet/met needs for those in state 2 (met health needs) at the estimated age.

LEs, life expectancies; multi-N, no multimorbidity; multi-P, physical multimorbidity; multi-PM, physical–mental multimorbidity; TLEs, total life expectancies.

## Discussion

The research is, to our knowledge, the first study globally to explore transitions in healthcare needs fulfilment (onset/out of unmet needs) and the effect of multimorbidity on these transitions. We found that those living with multimorbidity had higher 2-year probabilities of transitioning into unmet healthcare needs and having persistent unmet needs in two consecutive waves than the group without multimorbidity. P-morbidity and PM-multimorbidity were associated with increased risks of transitioning into unmet healthcare needs and with a reduced risk of transitioning out from unmet needs. Although women had longer estimated LEs than men, they had a higher rate of transitioning into unmet healthcare needs and had larger proportions of years spent with unmet needs. Higher education levels contributed to an increased likelihood of transitioning out of unmet healthcare needs, and the better-educated group had a lower proportion of time spent with unmet needs.

Although China has achieved substantial progress towards the UHC,^14^ there is a remaining gap in healthcare delivery that notably neglects the health needs of those living with multiple health conditions, many of whom are older people. Our findings suggest that multimorbidity acts as a crucial risk indicator in exacerbating the progression of unmet healthcare needs and hindering remission. This is consistent with previous findings on the association between multimorbidity and increased outpatient visits^11^ and a higher likelihood of having unmet health needs.^1^ Indeed, healthcare provision in different departments and facilities is often poorly coordinated for multiple health conditions, leading to more challenges in disease management and higher costs among the underserved population.^9^ We also observed that PM-multimorbidity and P-multimorbidity are comparable risk factors of transition into unmet health needs, implying that health services design must shift from solely focusing on physical conditions to addressing the diverse needs of managing PM-multimorbidity. In addition, there was no statistical evidence of the links between increasing age and transitioning into/out of unmet health needs after adjustment for social and health-related factors, which was partially supported by previous cross-sectional evidence in China.^8^ The attenuated effects of increasing age on transitions of health needs fulfilment could be attributed to chronic health conditions profiles as well as the complexity of health needs fulfilment (eg, the availability, affordability and acceptability of healthcare, etc).^4 5^

Our results identify higher educational levels as a predictor in the transition out of unmet healthcare needs. As a common social determinant, the association between education and well-being^25^ and healthy ageing^26^ have been broadly recognised. Health literacy potentially plays a mediating role between educational background and health-seeking behaviours.^27^ It may also independently modify upstream drivers in health that facilitate healthcare use, patient–clinician communication, and self-care^28^ and subsequently affect health downstream.^29^ In this study, compared with those in lower educational levels, the higher educated group had longer estimated LEs and were predicted to consistently spend less time having unmet needs at 60 years. Our results highlight the necessity of advocating health promotion interventions on enhancing health literacy around ageing and disease, as well as the potential benefits of improving the accessibility of services and better disease management.^30 31^

Consistently, our analyses of marginal LEs show that the proportion of time spent having unmet healthcare needs progressively increased with growing age with no gender differences, and regardless of multimorbidity status. The phenomenon is potentially a result of and a driver of inequalities in healthcare, as barriers to affordability, accessibility and acceptability of health services are often present among older people living in LMIC settings.^2^ Besides, our gender-specific findings are consistent with prior evidence on sex differences in health-seeking behaviour and service use in older Chinese^32^ and in disability-free and dependence-free LEs^33^; and indicate that women had longer LEs than men, but with a higher proportion of years spent living with unmet healthcare needs. The underlying mechanisms of these variations are supported by the sex differences in healthcare utilisation,^34^ with a trend of delays in treatment-seeking.^35^ Gender-specific barriers to multimorbidity management need to be warranted in further qualitative and interventional investigations to inform coping strategies.

Given our findings, multimorbidity was associated with shortened TLEs and led to longer marginal LEs with unmet needs. This is consistent with previous evidence on burdens in managing multiple long-term conditions, for example, increasing barriers to accessing healthcare across medical departments, polypharmacy issues, increased health expenditures, etc.^36 37^ The complexity of multiple chronic illness patterns raises more uncertainty in healthcare needs fulfilment and may result in the onset of unmet needs. Moreover, our estimations highlight that the proportions of years spent with having unmet healthcare needs over TLEs were higher in those with P-multimorbidity, followed by PM-multimorbidity in all estimated ages. This echoes that treatments for multimorbidity are inadequately designed and often delivered solely, lacking a continuum of integrated health services.^2 38^ Therefore, people with multiple conditions are poorly served, ultimately leading to unmet needs and worsening health and premature mortality. In our state-specific LEs estimates, having unmet needs as the starting state consistently predicted longer years spent with having unmet needs. This suggests that there is a cumulative effect of unmet health needs experiences through the path of health status, financial situation, and accessible information/resources, perhaps with deteriorating health leading to further unmet needs, from which it is difficult for individuals and households to be extricated. Impoverishment is likely to both result from and worsens this process.^39^

Research on transitioning into/out of meeting health needs is scarce. This is the first attempt to shed light on the transitions into and out of having unmet needs while simultaneously taking into account mortality. Based on nationally representative longitudinal data, this work provides robust estimations on the effect of time-varying multimorbidity on the transitions in met/unmet needs and death over 4 years of follow-up. Another key strength is the inclusion of parametric MSM, allowing robust estimates of total, marginal and state-specific LEs. However, some limitations remain in the current estimates. The findings are limited by any uncaptured transitions within the observation intervals and the nature of resourced measures on current unmet healthcare needs. The measure of unmet healthcare needs is generated by combining the unmet inpatient or outpatient care needs as a whole, but responses were based on retrospective experiences lacking clinical validation. Although this measure fits well with the conceptualisation of unmet healthcare needs,^4 18^ the approach may bias current MSM survival analyses on transitions and thereby affect the estimated LEs. In the estimation of LEs by multimorbidity, we considered baseline multimorbidity in the fitted MSM under the assumption of piecewise-constant approximation, as the ELECT estimates only allow for time-independent covariates.^22^ Sensitivity analyses for MSM with the same set of covariates and baseline multimorbidity suggest similar effects on transitions compared with the estimates of time-varying multimorbidity (table 2 and online supplemental table S4). Although we expect that multimorbidity status would not vary significantly within 2-year time intervals, there is still some uncertainty as to how this could affect the current results. For example, limitations may lie in the total and marginal LEs by baseline multimorbidity, as the estimates have not accounted for the changes in multimorbidity, which to some extent may affect the generalisability of findings. In addition, although a wide range of chronic conditions was captured and grouped into a 3-categorical multimorbidity by adopting a widely applied approach in health research,^9^ this study only captured a small range of mental health conditions that may be potentially experienced alongside physical illnesses. We cannot rule out the possibility of bias—for example, loss of information in grouping individual health conditions—which may affect our current estimates. Future research using a more theoretical approach to identify the different patterns of multimorbidity in health records data may provide additional validation of our findings. Finally, we acknowledge that the modelling does not account for survey weights due to the data structure restriction, along with losses of follow-up in a longitudinal study, probably impacting the representativeness of CHARLS for the Chinese population and possibly introducing selection bias to the current estimates. For example, the differences between our estimated TLEs at age 60 and official estimates from the World Bank^40^ may be due to the missingness of health needs states and sample attrition in follow-up. Moreover, it is possible that attrition is more concentrated in unmet needs groups, and so life expectancies with unmet needs may be subject to the attrition bias.^41 42^ However, the current longitudinal analysis still provides rich evidence on the profile of unmet/met healthcare needs transitions and death and the impact of multimorbidity in ageing Chinese populations.

## Conclusions

In summary, findings indicate that multimorbidity can potentially increase the risk of onset of unmet healthcare needs and hinder remission. The current health system is burdened by epidemiological and demographic transitions, highlighting the need for further policy inputs to better address the increasingly complex health needs of older people in relation to the management of physical–mental multimorbidity. It is crucial to fit the complex geriatric needs into further service solutions and integrations in order to bridge the enlarging gap in the current health system. Health literacy is a potentially modifiable component which could be addressed as a means of reducing unmet health needs. Further research is needed to provide insights into the effectiveness and feasibility of interventional packages for alleviating unmet healthcare needs and service scale-up. Although gaps remain in strengthening the primary healthcare system, it is vital to unpack trials on integrating chronic and long-term health needs derived from multimorbidity into primary care platforms. A delivery of personal-centred integrated healthcare that addresses the diverse health needs of older people with multimorbidity is fundamental for moving forward to better achieving the Sustainable Development Goals 3.4 and UHC.

## Data Availability

Data are available upon reasonable request. The CHARLS data were obtained available through the CHARLS website (http://charls.pku.edu.cn/en).
