# Supplementary material for: Multistate survival modelling of multimorbidity and transitions across health needs states and death in an ageing population
Source: J Epidemiol Community Health. 2024 Jan 11;78(4):212–9. doi: 10.1136/jech-2023-220570 (PMC10958265; doi:10.1136/jech-2023-220570)
Supplement: Supplementary data [file jech-2023-220570supp001.pdf]

Supplementary materials for: Multistate survival modelling of multimorbidity and transitions across health needs states and death in an ageing population

Supplementary Table S1. Full list of measured chronic illnesses in the study

Supplementary Table S2. Characteristics of study samples across waves

Supplementary Table S3. Numbers of transitions across states of healthcare needs and death

Supplementary Table S4. Effect of covariates on transitions aHRs (95% CI)

Supplementary Figure S1. Model for transitions across states of met and unmet healthcare needs and death

Supplementary Figure S2. A three-state model with the effect of 5-year age on transitions (HRs, 95%CI)

Gao Q, *et al. J Epidemiol Community Health* 2024; 78:212–219. doi: 10.1136/jech-2023-220570

Supplementary Table S1**.** Full list of measured chronic illnesses in the study

**Physical health conditions (n=12)**	**Mental health conditions (n=3)**
1) Hypertension<br>2) Dyslipidaemia<br>3) Diabetes or high blood sugar<br>4) Cancer or malignant tumour (excluding minor skin cancers)<br>5) Chronic lung diseases<br>6) Liver disease (excluding fatty liver, tumours, or cancer)<br>7) Heart attack, coronary heart disease, angina, congestive heart failure, or other heart problems<br>8) Stroke<br>9) Kidney disease (except for tumour or cancer)<br>10) Stomach or other digestive diseases (except for tumour or cancer)<br>11) Arthritis or rheumatism<br>12) Asthma	1) Emotional, nervous, or psychiatric problems<br>2) Memory-related disease (including Alzheimer's disease, Parkinson's disease, and cerebral atrophy)<br>3) Depression (measured by the 10-item Centre for Epidemiological Studies Depression Scale)

Gao Q, *et al. J Epidemiol Community Health* 2024; 78:212–219. doi: 10.1136/jech-2023-220570

Supplementary Table S2**.** Characteristics of study samples across waves

**Characteristics**	Baseline (N=14 156)	Wave 2 (N=18 153)	Wave 3 (N=19 532)	Multistate model sample (N=18 075)
**Interviewed (*N*)**	14 156	17 732	19 160	17 397
**Deaths (*N*)**	-	421	372	678
**Observations (*N*)**	14 156	17 732	19 160	46 646
**Health needs**				
Unmet healthcare needs	13.2%	13.3%	13.8%	13.5%
Met healthcare needs	86.8%	45.7%	70.7%	66.3%
Missingness (censored)	-	41.0%	15.5%	20.2%
**Age (SD)**	59.5 (9.8)	60.6 (9.9)	61.2 (10.2)	61.1 (9.9)
**Gender**				
Male	50.0%	48.8%	49.3%	49.1%
Female	50.0%	51.2%	50.7%	50.9%
**Education**				
Elementary school and below	65.8%	65.9%	69.2%	67.0%
Middle school	21.2%	21.0%	19.2%	20.9%
High school and above	13.0%	13.1%	11.5%	12.1%
Missingness	<0.1%	<0.1%	0.1%	<0.1%
**Multimorbidity**				
PM-multimorbidity	9.7%	7.9%	7.1%	8.3%
P-multimorbidity	37.1%	42.4%	49.4%	45.6%
None	53.2%	48.6%	43.0%	45.7%
Missingness	<0.1%	1.1%	0.5%	0.4%
**Multimorbidity at baseline**				
PM-multimorbidity	9.7%	9.5%	9.0%	9.7%
P-multimorbidity	37.1%	37.9%	34.0%	37.9%
None	53.2%	51.3%	55.9%	52.3%
Missingness	<0.1%	1.3%	1.1%	<0.1%

Note. Abbreviations: P-multimorbidity: physical multimorbidity; PM-multimorbidity: Physical-mental multimorbidity.

Supplementary Table S3. Numbers of transitions across states of healthcare needs and death

to<br>from	***State1:*** Unmet healthcare needs	***State2:*** Met healthcare needs	***State3:*** Death	***State -1*** [a]**:** Censored
State1: Unmet healthcare needs	995	1719	122	1051
State2: Met healthcare needs	2067	11 151	440	5215
State -1: Censored	810	4445	116	1118

a. State -1: Alive but with unknown last state

Gao Q, *et al. J Epidemiol Community Health* 2024; 78:212–219. doi: 10.1136/jech-2023-220570

Supplementary Table S4. Effect of covariates on transitions aHRs (95% CI)

**Transition I** *Transition out of unmet needs*		**Transition II** *Transition into unmet needs*	
Age	1.01 (1.00, 1.02)	Age	1.00 (0.99, 1.01)
Female (ref. male)	1.08 (0.95, 1.24)	Female (ref. male)	1.24 (1.08, 1.43)
Middle Education (ref. low)	1.12 (0.94,1.34)	Middle Education (ref. low)	0.95 (0.79, 1.14)
High education (ref.no)	1.32 (1.03, 1.71)	High education (ref.no)	1.22 (0.93,1.58)
P-multimorbidity (ref. no)	0.57 (0.49, 0.66)	P-multimorbidity (ref. no)	1.84 (1.58, 2.14)
PM-multimorbidity (ref. no)	0.68 (0.55, 0.85)	PM-multimorbidity (ref. no)	1.48 (1.18, 1.87)
**Transition III** *Unmet healthcare needs- Death*		**Transition IV** *Met healthcare needs- Death*	
Age	1.05 (1.01, 1.09)	Age	1.11 (1.10, 1.13)
Female (ref. male)	0.39 (0.19, 0.80)	Female (ref. male)	0.79 (0.64, 0.98)
Middle Education (ref. low)	1.80 (0.86, 3.76)	Middle Education (ref. low)	0.85 (0.58, 1.24)
High education (ref.no)	1.22 (0.48, 3.10)	High education (ref.no)	0.40 (0.21,0.75)
P-multimorbidity (ref. no)	3.29 (0.87, 12.45)	P-multimorbidity (ref. no)	1.65 (1.32, 2.07)
PM-multimorbidity (ref. no)	5.98 (1.49, 23.93)	PM-multimorbidity (ref. no)	1.15 (0.77, 1.71)

Note. Abbreviations: aHR, adjusted Hazard ratios; 95%CI, 95% confidence intervals. P-multimorbidity, physical multimorbidity; PM-multimorbidity, Physical-mental multimorbidity. High-education: high school and above; Middle-education: middle school; Low-education: elementary school and below.

The model adjusted for age, sex, education, and baseline multimorbidity (Model fit: -2loglik = 22470.03; AIC = 22526.03)

Supplementary Figure S1. Model for transitions across states of met and unmet healthcare needs and death

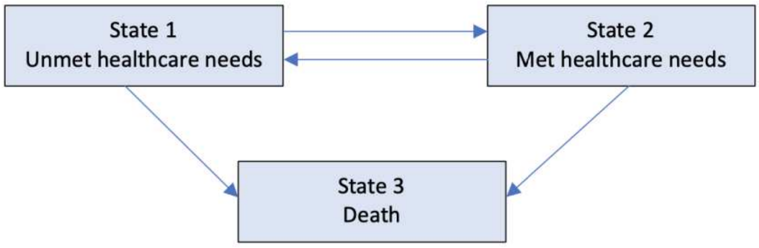

Supplementary Figure S2. A three-state model with the effect of 5-year age on transitions (HRs, 95%CI)

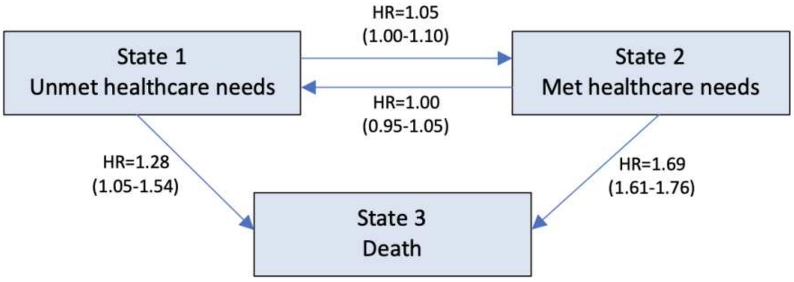

Note. Abbreviations: HR, Hazard ratios; 95% CIs, 95% confidence intervals.

The estimates were based on multistate survival model (adjusted for age, sex, education, and time-varying multimorbidity).
